# Implementation and evaluation of an end‐to‐end IGRT test

**DOI:** 10.1120/jacmp.v13i5.3939

**Published:** 2012-09-06

**Authors:** Stephen F. Kry, Jimmy Jones, Nathan L. Childress

**Affiliations:** ^1^ Department of Radiation Physics The University of Texas MD Anderson Cancer Center Houston Texas; ^2^ The Methodist Hospital Mobius Medical Systems Houston Texas USA; ^3^ LP Houston Texas USA

**Keywords:** IGRT, Winston‐Lutz, image‐guided, setup error, positioning, imaging

## Abstract

The goal of this work was to develop and evaluate an end‐to‐end test for determining and verifying image‐guided radiation therapy setup accuracy relative to the radiation isocenter. This was done by placing a cube phantom with a central tungsten sphere directly on the treatment table and offset from isocenter either by 5.0 mm in the longitudinal, lateral, and vertical dimensions or by a random amount. A high‐resolution cone‐beam CT image was acquired and aligned with the tungsten sphere in the reference CT image. The table was shifted per this alignment, and megavoltage anterior–posterior and lateral images were acquired with the electronic portal imaging device. Agreement between the radiation isocenter (based on the MV field) and the center of the sphere (i.e., the alignment point based on kV imaging) was determined for each image via Winston‐Lutz analysis. This procedure was repeated 10 times to determine short‐term reproducibility, and then repeated daily for 51 days in a clinical setting. The short‐term reproducibility test yielded a mean 3D vector displacement of 0.9±0.15mm between the imaging‐based isocenter and the radiation isocenter, with a maximum displacement of 1.1 mm. The clinical reproducibility test yielded a mean displacement of 1.1±0.4mm with a maximum of 2.0 mm when the cube was offset by 5.0 mm, and a mean displacement of 0.9±0.3mm with a maximum of 1.8 mm when the cube was offset by a random amount. These differences were observed in all directions and were independent of the magnitude of the couch shift. This test was quick and easy to implement clinically and highlighted setup inaccuracies in an image‐guided radiation therapy environment.

PACS numbers: 87.55.km; 87.55.Qr; 87.56.Fc

## I. INTRODUCTION

Image‐guided radiation therapy (IGRT) has become a staple of modern radiotherapy. An integral part of the IGRT process is the coincidence of the imaging isocenter with the radiation treatment isocenter. There will always be a discrepancy between these two isocenters because they are associated with different mechanical systems. This isocenter coincidence is also affected in clinical practice by the IGRT workflow, including imaging, target alignment that is often software‐based, and couch motion.

Verification of the coincidence of the imaging isocenter and radiation isocenter, verification of the software based‐alignment, and verification of correct couch shifts are all integral to modern IGRT quality assurance (QA).^(1)^ Hence, numerous tests have been proposed to address the QA of IGRT systems, most notably by Yoo et al.,^(2)^ but also by many others.^3^, ^8^ The majority of IGRT QA tests in the literature involve aligning a phantom with external lasers (or crosshairs), imaging the phantom, and noting any discrepancy between the imaging isocenter and the center of the phantom.

However, there are three major drawbacks to this type of test. First, instead of testing alignment of the imaging isocenter with the actual radiation treatment isocenter, it tests alignment of the imaging isocenter with a surrogate for the radiation isocenter, usually the crosshairs. Although agreement of the crosshairs and the radiation isocenter can be separately evaluated and maintained, this practice introduces uncertainty and reduces efficiency. Second, this approach does not mimic the clinical workflow of an IGRT case, in which the imaging system, not the lasers, is used to align the target. Third, such an approach does not allow an end‐to‐end evaluation of the IGRT process.

Therefore, we propose and evaluate an end‐to‐end test that mimics clinical IGRT workflow and verifies final target alignment (based on imaging) with the radiation treatment isocenter. This approach combines an end‐to‐end test (as proposed by Guan et al.^(6)^) while avoiding the use of room lasers (as proposed by Du et al.^7^, ^9^) by scanning, aligning, and shifting a cube phantom and verifying isocenter alignment using a Winston‐Lutz test. Because this is an end‐to‐end test, it can also aid the assessment of the entire clinical IGRT process, including computed tomography (CT) performance, placement of the isocenter in the planning system, transfer of coordinates from CT to the linear accelerator through the planning system, couch motion, imaging alignment, beam collimation (with either jaws or a multileaf collimator), and coincidence of the imaging isocenter with the radiation isocenter. However, the necessary parts of this test (as described by the requirements of the Task Group 142 report (TG‐142)^(1)^) are simple and can be conducted daily from start to finish in less than 10 minutes. Simple expansions beyond these requirements can allow the evaluation of numerous aspects of the end‐to‐end IGRT process.

## II. MATERIALS AND METHODS

### A.1 Procedure

We created an end‐to‐end QA test that mimics the traditional workflow of IGRT‐based treatments.

**Figure 1:**
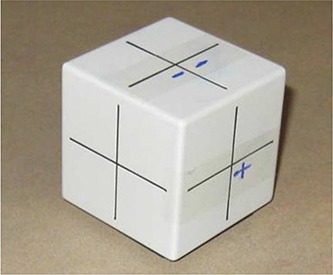
Custom plastic cube phantom used for IGRT tests measuring 5 cm per side with an 8 mm tungsten ball in the center.

This test used a custom cube phantom (Modus Medical Devices, Inc., London, Ontario, Canada) consisting of a tungsten sphere 8 mm in diameter embedded at the center of a $5\;{\text{cm}}^{3}$ plastic cube (Fig. 1). The plastic surrounding the tungsten sphere was scribed with marks indicating the center of the tungsten sphere for reproducible visual alignment. Off‐center markings (5 mm from the center marks in each of the vertical, lateral, and longitudinal directions) were also present for use in setups where a displacement from the isocenter was desired.

The first half of the test assessed the transfer of information. The cube phantom first underwent a CT scan with a 1 mm slice thickness using a clinical simulator (Brilliance big bore; Philips Healthcare, Andover, MA). The images were then exported to a treatment planning system (Pinnacle^3^; Philips Healthcare). The central tungsten sphere was contoured, and isocenter was placed at its centroid. Fields of $2\times 2{\;\text{cm}}^{2}$ defined by the multileaf collimator were then added in the anterior–posterior (0°) and lateral (270°) orientations. These fields were transferred to the Record and Verify system.

**Figure 2:**
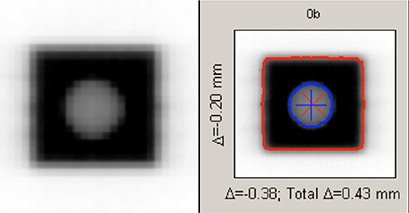
A sample Winston‐Lutz image and analysis. The left panel is a raw image from the electronic portal imaging device. The right panel shows analysis of the Winston‐Lutz image, including differences (Δ) between the center of the radiation field and the tungsten ball centroid.

The second half of the test assessed imaging and alignment. The cube phantom was placed on the treatment couch either at a random location or aligned with a set of marks. The phantom was scanned with a cone beam CT at maximum resolution (512×512 matrix with a 1 mm slice thickness). This image was aligned with the reference image using 3D‐3D alignment on a Varian 4D Integrated Treatment Console (Varian Medical Systems, Inc., Palo Alto, CA), such that the centroid of the tungsten sphere in the acquired image was aligned with the imaging isocenter. Automatic couch motions were then used to shift the phantom to the imaging isocenter and the cube was then irradiated with the $2\times 2{\;\text{cm}}^{2}$ Winston‐Lutz fields onto the EPID. A source‐to‐imager distance of 150 cm was used to produce a relatively small pixel size of $0.26\times 0.26\;{\text{mm}}^{2}$ at the isocenter. The resultant A‐P and lateral images (see Fig. 2) were then analyzed on the local computer to determine how well the imaging isocenter aligned with the radiation isocenter. All Winston‐Lutz analyses were conducted in DoseLab Pro (Mobius Medical Systems, LP, Houston, TX) using its automatic Winston‐Lutz analysis package. This analysis tool uses area averaging that allows for a resolution of < 0.1 mm in determining the centroid of the sphere and radiation isocenter.

### A.2 Evaluation

The procedure described above was used under several conditions to evaluate the reproducibility of its results, as well as the precision of a standard IGRT regimen. Because the focus of IGRT QA is on imaging and alignment, the second half of the test (imaging and alignment) was the focus of our evaluation.

First, the consistency of the imaging system and the Winston‐Lutz analysis was assessed. The cube phantom was placed on the table in alignment with the room lasers. Ten successive images were taken with the $2\times 2{\;\text{cm}}^{2}$ field at each of the linear accelerator's cardinal angles.

On each of the 40 images, the distance from the centroid of the sphere to the center of the radiation field was determined (Fig. 2). As the phantom was not moved, the standard deviation and range of this difference assessed the reproducibility of the EPID imaging and the Winston‐Lutz analysis as performed in this test.

Second, the short‐term reproducibility of the performance of an IGRT system was assessed for two different Varian 2100 iX linear accelerators (Varian Medical Systems). For each linear accelerator, the phantom was offset from isocenter by a random distance in a random 3D direction. The phantom was then imaged via cone‐beam CT, aligned with the centroid of the sphere using 3D‐3D alignment, shifted by way of automatic couch motion, and then A‐P and lateral Winston‐Lutz images were taken using the $2\times 2{\;\text{cm}}^{2}$ fields. These images were analyzed to determine the residual difference between the centroid of the sphere (corresponding to the alignment based on the imaging isocenter) and the center of the radiation field (corresponding to the treatment isocenter). The phantom was then manually moved from the isocenter location, and this process was repeated 10 times in succession for each linear accelerator (20 times total).

Third, the clinical reproducibility of this procedure and a standard IGRT protocol were assessed. A certified radiation therapist conducted the imaging and alignment portion of the test twice each morning for a period of 51 successive days. Two different initial offsets were assessed each day. First, the phantom was aligned to the off‐center marks with room lasers (offset from the center of the tungsten sphere by 5.0 mm in each direction). The phantom then underwent cone‐beam CT, 3D‐3D alignment, automatic couch shifting, and exposure to the A‐P and lateral $2\times 2{\;\text{cm}}^{2}$ fields. This process was then repeated with the phantom randomly offset from isocenter by 2.0–23.0 mm in each direction. In total, the therapist generated 102 unique alignments with 204 unique Winston‐Lutz images. Statistical analysis of the clinical reproducibility data was conducted with unpaired t‐tests to compare the impact of a random initial alignment offset versus a preset offset, and to compare the impact of the size of the initial alignment offset. Finally, linear regression analysis was conducted to assess if the residual alignment error (the difference between the centroid of the sphere and the center of the radiation field following IGRT alignment) changed during the 51 days over which measurements were taken.

## III. RESULTS

The transfer of information portion of the end‐to‐end test required less than 60 minutes to scan the phantom, contour the target, define an isocenter, place treatment fields, and transfer the plan to the Record and Verify system. The imaging and alignment portion of the test, including cone‐beam CT, alignment, and analysis of the Winston‐Lutz images, required approximately 10 minutes for either the therapist or physicist. These steps provided a relatively quick end‐to‐end assessment of the IGRT procedure, following a clinical IGRT workflow.

The results of the consistency test, in which repeated exposures were taken without moving the phantom between exposures, are shown in Table 1. Very consistent results were observed; the standard deviation of the difference between the center of the sphere and the center of the radiation field was ≤ 0.03 mm in all directions for all gantry angles. The range of this difference was also small, the maximum being 0.12 mm. These results indicate that the EPID imaging system and the Winston‐Lutz test are stable and reproducible.

**Table 1:** Residual difference between IGRT‐aligned isocenter (tungsten sphere centroid) and radiation field isocenter. Data are results of 10 sequential exposures of the phantom (no movement between exposures). Range is the absolute value of the difference between the maximum and minimum values for each set of exposures at each angle.

	*Gantry Angle*							
	*0* ^*0*^		*90* ^*0*^		*180* ^*0*^		*270* ^*0*^	
*Value*	*LAT (mm)*	*LNG (mm)*	*VRT (mm)*	*LNG (mm)*	*LAT (mm)*	*LNG (mm)*	*VRT (mm)*	*LNG (mm)*
Standard Deviation	0.03	0.03	0.02	0.01	0.03	0.03	0.01	0.02
Range	0.08	0.11	0.08	0.02	0.09	0.12	0.04	0.04

LAT: lateral dimension; LNG: longitudinal dimension; VRT: vertical dimension.

The results of the assessment of short‐term reproducibility (repeated exposures while moving the phantom between exposures) are shown for each of the two linear accelerators in Table 2. The mean displacements in each direction for linear accelerator 1 were close to 0. This indicates that, on average, the cone‐beam CT and alignment system moved the cube phantom to the radiation isocenter. For linear accelerator 2, the average difference between the imaging and radiation isocenters was less close to 0, indicating slight disagreement between the imaging and radiation isocenters. Regardless, for both linear accelerators, there was always error in each individual alignment. The maximum alignment error in each direction was 0.5–0.9 mm, and the standard deviation, although typically small, was as high as 0.7 mm. For both linear accelerators, the overall 3D alignment error was 0.9 mm on average, with a maximum of 1.1 mm. The uncertainties in the mean residual errors in each direction (as measured by the standard deviation of the mean) were small, averaging only 0.15 mm.

**Table 2:** Residual difference between IGRT‐aligned isocenter (tungsten sphere centroid) and radiation field isocenter, along with the standard deviation and magnitude of the maximum difference. Data are results of 10 IGRT‐positioned phantom exposures conducted with two different linear accelerators for a total of 20 exposures. The standard deviation of the mean (standard error) for both linear accelerators was 0.15 mm.

	*AP Image*		*LAT Image*		
*Accelerator*	*LAT (mm)*	*LNG (mm)*	*VRT (mm)*	*LNG (mm)*	*Total 3D vector*
1 Mean	0.0	0.0	0.0	0.5	0.9
Std. Deviation	0.7	0.4	0.4	0.4	0.2
Max	0.9	0.9	0.7	0.9	1.0
2 Mean	0.4	0.3	0.6	0.7	0.9
Std. Deviation	0.3	0.2	0.1	0.2	0.1
Max	0.7	0.5	0.7	0.9	1.1

LAT: lateral dimension; LNG: longitudinal dimension; VRT: vertical dimension.

The longitudinal alignment of the sphere and radiation field was evaluated in both the anterior–posterior image (0°) and the lateral image (270°). The mean residual alignment error (the distance between the center of the sphere and the center of the radiation field following IGRT alignment) averaged over both linear accelerators was 0.15 mm on the anterior–posterior image, but 0.6 mm on the lateral image. This difference between the two images of nearly 0.5 mm shows that the radiation isocenter changes with gantry angle (i.e., gantry sag). Such change can be an additional confounding factor in multi‐angle alignment and treatment; other investigators have also noted deviations of up to 1.5 mm in the imaging isocenter at different gantry angles.^10^, ^11^ Other than this systematic offset, the remaining residual error was random, and similar maximum residual errors were seen in the lateral, longitudinal, and vertical dimensions.

**Figure 3:**
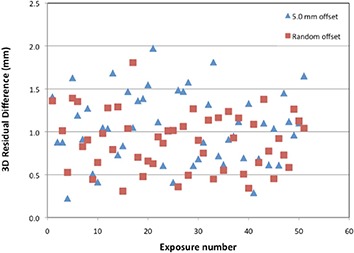
The residual difference between the IGRT‐aligned isocenter (the tungsten sphere centroid) and radiation treatment isocenter for 51 days of Winston‐Lutz analysis. Each day the phantom was imaged twice, once with an initial setup offset of 5.0 mm from isocenter, and one with a random offset.

The results of the clinical reproducibility test are presented in Fig. 3. This figure shows the residual alignment error between the radiation isocenter and the IGRT‐based isocenter for the 204 Winston‐Lutz tests. In this case, the residual error is calculated as the magnitude of the 3D vector difference. These data, summarized in Table 3, show findings similar to those of the short‐term reproducibility tests (Table 2). Sizeable random differences between the radiation isocenter and the imaging‐based isocenter were seen on a day‐to‐day basis. The average residual 3D difference for all setups was 1.0 mm; however, the maximum residual difference was 2.0 mm.

**Table 3:** Residual difference between IGRT‐aligned isocenter (tungsten sphere centroid) and radiation field isocenter, along with the standard deviation and magnitude of the maximum difference. Data are results of 51 IGRT‐positioned phantom exposures. The phantom was set up daily with either a 5.0 mm offset from the isocenter or a random offset of 2.0–23.0 mm in each dimension. The standard deviation of the mean (standard error) of the 3D vector was 0.08 mm.

		*AP Image*		*LAT Image*		
*Offset Amount*		*LAT (mm)*	*LNG (mm)*	*VRT (mm)*	*LNG (mm)*	*Total 3D vector*
5.0 mm	Mean	0.5	0.2	0.1	0.7	1.1
	Std. Deviation	0.5	0.7	0.4	0.7	0.4
	Max	1.5	1.5	0.5	1.9	2.0
Random	Mean	0.1	0.2	0.0	0.6	0.9
	Std. Deviation	0.5	0.6	0.5	0.6	0.3
	Max	1.0	1.5	0.7	2.0	1.8

LAT: lateral dimension; LNG: longitudinal dimension; VRT: vertical dimension.

We conducted additional analyses on the clinical reproducibility data. First, we compared the residual 3D alignment error for the phantom setups with an initial 5.0 mm offset with those for setups with a random initial offset. The mean residual error was 1.1 mm (SD=0.4 mm) with the 5.0 mm offset and 0.9 mm (SD=0.3 mm) with the random offset. This 0.2 mm difference was statistically significant (p=0.04), but is small and not clinically significant. Therefore, this test can be performed with either alignment method. Second, for the random initial offset setups, we compared the residual 3D alignment error in setups with an offset of > 10 mm and those with an offset of ≤ 10 mm; no significant difference was found (p=0.5). This is important in clinical IGRT, as it indicates that the ability of the IGRT system to align the target to the radiation isocenter is independent of the magnitude of the shift. Indeed, many of the smallest alignment shifts (e.g., 5 mm) were associated with > 1 mm of residual error. Finally, regression analysis was done to determine if the number of days over which this test was performed impacted the residual 3D error (from day 1 to day 51). In general, this would be a test of the stability of the imaging isocenter versus the radiation isocenter; an increase in magnitude of the 3D error with time could indicate degradation of the alignment system. In the current experiment, this test also would have indicated whether proficiency in aligning the phantom increased with time (which would have appeared as decreasing setup error with time), or whether attentiveness to the setup procedure decreased with time (increasing setup error with time). We found that the residual alignment error over all 102 tests did not significantly change with time (5mm offset: slope =‐0.0009 mm/day, p=0.8; random offset: slope=‐0.0023mm/day, p=0.5). Therefore, the operators of the test, the test itself, and the machine being examined were stable over the two months that these tests were conducted.

## IV. DISCUSSION

We developed a test that follows and evaluates the end‐to‐end workflow of clinical IGRT. The transfer portion of this test (including initial scanning and contouring) required approximately 60 minutes, and the imaging and alignment phase required less than 10 minutes. This test was successfully implemented, and sample results were evaluated to provide clinical insight into the precision of the IGRT process.

The results of the average alignment errors (Tables 2and 3) showed that although the imaging and radiation isocenters generally agreed, residual error remained after IGRT setup. The center of the target did not move precisely to the radiation isocenter, partly because gantry sag moved the radiation isocenter with changes in gantry angle, but more substantially because the automatic couch shifts did not precisely align the center of the target with the radiation isocenter. This error, as a 3D vector, was 1 mm on average but could be as large as 2 mm. This error was independent of the size of the couch shifts and was of similar magnitude in all directions. The magnitude of this residual error was consistent with the couch shift error reported by Yoo et al.,^(2)^ although their findings did not show large residual errors in the lateral dimension, as our study did. Sharpe et al.^(12)^ found residual positioning errors of up to 2 mm, also comparable to our findings. They attributed their major alignment discrepancies to couch positioning accuracy, which is consistent with our findings — even with excellent alignment between imaging and radiation isocenters (Table 2), there were sizable residual errors in the final alignment of a target. This is of note when considering TG‐142, which specifies equal precision for the alignment of radiation versus imaging isocenters and the ability of the system to position/reposition. Indeed, the latter procedure (position/reposition) is required to be ≤ 1 mm for SRS/SBRT systems. The present work calls into question the feasibility of achieving this level of precision based on QA performed according to clinical workflow.

In both the short‐term reproducibility and the clinical reproducibility tests, the results indicated both random and systematic errors in the agreement between imaging and radiation isocenters. As an end‐to‐end test, this test detects errors introduced from numerous sources, including misalignment of the radiation versus imaging isocenters, couch movement errors, imaging system misalignments and, because it is based on a Winston‐Lutz test, centering of the MLC about the radiation isocenter. The random sources of error were typically close to 1 mm and were generally larger than the systematic errors. Reducing the random errors would likely require improved couch driving precision. The systematic errors were attributable to the change in radiation isocenter with gantry angle, which cannot readily be improved, and incorrect alignment between the imaging and radiation isocenters, which can be resolved through careful definition of the imaging isocenter. Systematic errors revealed by this test were on the order of 0.5 mm (Table 2).

The results of this work highlight the important observation that congruence of the imaging and treatment isocenters still leaves up to 2 mm of residual alignment error in a clinical scenario. Even with perfect congruence of isocenters, it is not possible to avoid random error in a given clinical scenario (e.g., a given patient treatment). It is therefore necessary to include the residual alignment error in the definition of the planning target volume. An alignment margin of 1–2 mm is likely appropriate in clinical practice. With repeated imaging and alignment, larger errors (such as 2 mm alignment errors) may be noted, and a patient could be realigned to potentially reduce residual error. However, routine alignment within 1 mm is practically unattainable, even for rigid bodies.

In terms of implementation, this end‐to‐end test comprises two main components that test different aspects of the IGRT process. The initial component of setup and data transfer is primarily a test of the software involved in the IGRT process, and although this component is a critical part of the process, it is not prone to “drifting” out of tolerance and is not checked routinely when following TG‐142 guidance.^(1)^ Hence, this component of the test is generally important only when a complete end‐to‐end test is warranted, for example when new hardware or software is installed or replaced, or perhaps annually. The imaging and alignment portion tests the mechanical components of IGRT and the software that drives them. Such tests are recommended on a daily, monthly, and annual basis in TG‐142 and other QA documents pertaining to on‐board imagers.^2^, ^5^ In this study we found that a qualified radiation therapy technologist was able to complete the imaging and alignment component of the end‐to‐end test in 10 minutes. Therefore, this test is an easily implementable evaluation of the IGRT system.

## V. CONCLUSIONS

We have proposed and validated an end‐to‐end IGRT test that accurately and rapidly meets the requirements of TG‐142 and follows the workflow of clinical IGRT practice.

We found that even with congruence of the radiation and imaging isocenters, there was still a residual alignment error in the IGRT process of 1 mm on average and 2 mm at maximum. This residual alignment error occurred in all three dimensions, and was independent of the size of the initial couch shift. This residual error should be accounted for in clinical margin determination.
